# Analysis on the desert adaptability of indigenous sheep in the southern edge of Taklimakan Desert

**DOI:** 10.1038/s41598-022-15986-x

**Published:** 2022-07-18

**Authors:** Cheng-long Zhang, Chunjie Liu, Jihu Zhang, Langman Zheng, Qianqian Chang, Zilong Cui, Shudong Liu

**Affiliations:** 1 College of Animal Science and Technology, Tarim University, Alar, 843300 China; 2 Key Laboratory of Tarim Animal Husbandry Science and Technology, Xinjiang Production and Construction Corps, Alar, 843300 China

**Keywords:** Computational biology and bioinformatics, Genetics, Molecular biology

## Abstract

The southern margin of the Taklimakan Desert is characterized by low rainfall, heavy sandstorms, sparse vegetation and harsh ecological environment. The indigenous sheep in this area are rich in resources, with the advantages of perennial estrus and good resistance to stress in most sheep. Exploring the molecular markers of livestock adaptability in this environment will provide the molecular basis for breeding research to cope with extreme future changes in the desert environment. In this study, we analyzed the population genetic structure and linkage imbalance of five sheep breeds with three different agricultural geographic characteristics using four complementary genomic selection signals: fixation index (FST), cross-population extended haplotype homozygosity (xp-EHH), Rsb (extended haplotype homozygosity between-populations) and iHS (integrated haplotype homozygosity score). We used Illumina Ovine SNP 50K Genotyping BeadChip Array, and gene annotation and enrichment analysis were performed on selected regions of the obtained genome. The ovary of Qira Black sheep (Follicular phase, Luteal phase, 30th day of pregnancy, 45th day of pregnancy) was collected, and the differentially expressed genes were screened by transcriptomic sequencing. Genome-wide selective sweep results and transcriptome data were combined for association analysis to obtain candidate genes associated with perennial estrus and stable reproduction. In order to verify the significance of the results, 15 resulting genes were randomly selected for fluorescence quantitative analysis. The results showed that Dolang sheep and Qira Black sheep evolved from Kazak sheep. Linkage disequilibrium analysis showed that the decay rate of sheep breeds in the Taklimakan Desert was higher than that in Yili grassland. The signals of FST, xp-EHH, Rsb and iHS detected 526, 332, 308 and 408 genes, respectively, under the threshold of 1% and 17 overlapping genes under the threshold of 5%. A total of 29 genes were detected in association analysis of whole-genome and transcriptome data. This study reveals the genetic mechanism of perennial estrus and environmental adaptability of indigenous sheep breeds in the Taklimakan Desert. It provides a theoretical basis for the conservation and exploitation of genetic resources of indigenous sheep breeds in extreme desert environment. This provides a new perspective for the quick adaptation of sheep and other mammals to extreme environments and future climate changes.

## Introduction

Sheep have been domesticated for about 11,000 years^1^, providing humans with products such as meat, milk, skin and wool. Breed choice for sheep has been based on their production performance and reproductive capacity in grassland and plain areas, but less in a desert environment.

The Taklimakan Desert is located north of the Kunlun Mountains and has a harsh ecological environment^2^. This area is considered unfavorable to sheep breeding due to the high salinity, strong solar radiation, large temperature difference between day and night and forage shortage. Analyzing the adaptation of sheep breeds to extreme environment is of great significance for current livestock breeding and future climate changes.

Researchers have recently used female Dorset sheep from the BGI-Huada Genomics Institute and Chinese Academy of Sciences and published the sheep genome sequence in 2014. They used a combination of next-generation sequencing (NGS) and genome mapping technologies, such that the sequencing depth was 75X, and the full length of the resulting genome was 2.61 Gb^3^. The advancements in sheep genome sequencing provide convenient genetic information for the functional gene location, analysis and related molecular markers in sheep^4^. Li et al.^5^ applied a combination of genome-wide association analysis and selective cleaning on 99 Finnsheep populations of different colors to obtain the genes related to the coat color (TYRP, ASIP, MITF). Based on Illumina Ovine SNP 50K Genotyping BeadChip, Kijas et al.^6^ used the FST method to carry out selective cleaning among 74 sheep breeds to obtain genes related to pigment deposition (KIT, MITF), body size, muscle hypertrophy, hair quality and production traits (NPR2, HMGA2, BMP2, FGF5); they proved that the ASIP gene is related to the black and white hair color of the sheep. A selective sweep analysis between two different types of sheep fat tail breeds was performed by Moradi et al.^7^ based on the Illumina Ovine SNP 50K Genotyping BeadChip, and they found that fat-tailed sheep have selection signals in OAR5 and OARX, while lean-tailed sheep have selection signals in OAR7. In the work of Z. Yuan et al.^8^, two selective cleaning methods, FST and hapFLK, were used to analyze fat-tailed and lean-tailed sheep populations, and candidate genes related to tail traits were obtained, including HOXA11, BMP2, PPP1CC, SP3, SP9, PROKR1 and ETAA1. Wang and colleagues^9^ performed a genomic selective sweep analysis of three sheep breeds (Mongolian, German Mutton Merino, African white Dorper) and obtained genes related to body index (APOBP, GTO), meat quality traits (ALDOA, STK32B, FAM190A), reproductive traits (CCNB2, BXP2 and SLC8A3), hair quality traits (EDAR) and ear size (MSRB3). The Illumina Ovine SNP 50K Genotyping BeadChip was further used by Wei et al.^10^ on six breeds of sheep populations to divide them into two groups according to the living environment of different altitudes using FST and xp-EHH analysis; as a result, they obtained the adaptation-related candidate genes of EPAS1, CRYAA, LONP1, NF1, DPP4, SOD1, PPARG and SOCS2. Ji Yang^11^ divided Chinese sheep breeds into three groups: Yunnan-Kweichow, Qinghai-Tibetan and North-Eastern based on geographic location and obtained candidate genes that are adapted to different ecological environments (IFNGR2, MAPK4, NOX4, SLC2A4, PDK1, SOCS2, CALM2, CACNA2D1, KCNJ5 and COX2) based on the sheep genome resequencing technology. Wang et al.^12^ performed an analysis on Tibetan sheep, Altay sheep, Duolang sheep, Hu sheep and Mongolian sheep using the method of sheep genome resequencing, and found 22.425-22.575Mb on the OAR22 chromosome to be involved in the regulation of blood hemoglobin levels. The CYP17 gene is associated with the number of red blood cells and high-altitude polycythemia, while DNAJB5 can improve the cell tolerance to stress factors (most notably heat stress) on the OAR2 chromosome in Duolang sheep^13,14^. However, there is little research on the genetic mechanism behind the adaptability of sheep breeds in extreme desert environments.

With the progressive global warming and intensified desertification, extreme weather events occur frequently. Since the domesticated sheep breeds in temperate zone have poor environmental tolerance, some of them are endangered, and genetic resources are lost. Understanding the genetic mechanism behind the adaptation to different environments in sheep breeds can help to develop strategies to protect endangered breeds. In this work, we investigated the genetic mechanism of perennial estrus and environmental adaptability of indigenous sheep breeds in Taklimakan Desert by combining genome and transcriptome analysis of five sheep breeds with three different agro-geographical characteristics. This provides a theoretical basis for the development and protection of sheep germplasm resources in extreme desert environments.

## Materials and methods

### Ethics statement

All animals were handled according to the Guidelines for the Biological Studies Animal Care and Use Committee, People’s Republic of China. Animal experiments were approved by the Animal Ethics Committee of the College of Animal Science and Technology of Tarim University.

### Animal samples

A total of 314 healthy unrelated adult ewes belonging to five sheep breeds from three different agro-geographical zones were included. The sheep breeds were divided into three groups. The first group from Taklimakan Southeern Edge of Desert, including 36 Qira Black sheep (QR) from Qira Sheep Farm, Qira County, Xinjiang and 21 Duolang sheep (DL) from Maigaiti Sheep Farm, Maigaiti County, Xinjiang. The second group from Yili Grassland, inlcuding 66 Kazakh sheep (HSK) from Gongliu sheep Farm, Gongliu County, Yili, Xinjiang. The third group from the International Sheep Genomics Consortium (ISGC, http://www.sheephapmap.org), including 103 Dorset sheep (APD) and 88 Suffolk sheep (ASU). RNA-seq samples were provided by Luyuan Bio-technology Co., Hotan Prefecture, Xinjiang. We selected healthy Qira black sheep (QR) as well as ovarian tissues at follicular stage (FP), luteal stage (LP), 30 days of gestation (P30) and 45 days of gestation (P45). Three tissue samples were replicated in each period of RNA-Seq and qRT-PCR, and three biological replicates were performed in each experiment.

### Genotyping and quality control

We collected 0.25g tissue from the ear margin of sheep using ear pliers and placed the sample in a 1.5-mI sterilized centrifuge tube containing 75% alcohol. DNA was extracted using the phenol-chloroform method, and Ilumina Ovine SNP50 BeadChip was prepared. The Genome Studio software was used to process the basic data and obtain VCF files, while PLINK software v.1.905^15^ was used to evaluate the quality of single nucleotide polymorphism (SNP) genotype data and remove unqualified SNPs from the included samples. We used the following criteria: inclusion detection rate less than 90%, minimum allele frequency less than 5% and hardy-Weinberg balance test P value less than $1\times 10^{-6}$.

### Genetic diversity and population structure

Principal component analysis (PCA) was conducted on SNPs data after quality control using the software PLINK^15^. The VCF2Dis software (https://github.com/BGI-shenzhen/VCF2Dis) was used to calculate the P distance matrix, based on which a neighbor-joining phylogeny was constructed. The tree was visualized using the iTOL tool^16^. To perform admixture analysis, LD pruning was carried out in a sliding window of 50 SNPs with a step size of 10 SNPs by PLINK^15^. All SNPS with an $r^2$ of LD in each window exceeding the threshold of 0.1 (-indep-pairwise 50 10 0.1) were deleted, and the SNPs with the highest MAF in the PAIR of SNPs in LD were retained. After pruning, we selected the K value within the range 2–14 to perform population genetic structure analysis using Admixture^17^. In order to determine the most probable ancestral populations, the lowest cross-validation error of 14-fold was applied. LD (average value of $r^2$) between SNPs among varieties was estimated using the PopLDdecay software v3.41^18^, and the results were visualized using a Perl script.

### Selection sweep methods

In order to capture the genomic selection signals more accurately, more than one method is usually needed^19^. Therefore, we implemented four complementary statistical tests: FST (fixation index), xp-EHH (cross-population extended haplotype homozygosity), Rsb (extended haplotype homozygosity between-populations) and iHS (integrated haplotype homozygosity score).We performed Intra-population genomic selective sweep analysis using iHS on all individuals (QR and DL) in the first group, and pairwise comparison for (a) QR and DL versus HSK, and (b) QR and DL versus APD and ASU to identify genomic regions under increasing differentiation using FST, xp-EHH and Rsb.

The iHS method uses a single marker locus to replace the core haplotypes in EHH statistics, defining them as core loci. The ancestors in the core loci alleles in the haploid type are extended, and new mutant alleles in extension EHH statistics of haploid genetic distance integral are obtained, and the ratio between them is calculated to select the signal detection statistic.

FST analysis is a widely used method^20^ to identify genetic differences between populations compared with the frequency of polymorphism within the populations. The FST value represents the basic measure of genetic difference between two populations. We performed the FST analysis to identify genomic regions that are under increasing differentiation using VCFtools v0.1.15. For each comparison, the average FST value was calculated across all 45,298 SNPs.

The xp-EHH test^21^ can be used to perform a complete selective scan, by comparing the corresponding haplotype of each population with that of other populations.

The Rsb^22^ test is used to identify selective sweeps based on the same idea of estimating EHH as the in the xp-EHH test. However, unlike the xp-EHH test, Rsb does not require phasing information^23^. Similar to other statistics that usually focus on comparing genetic variation between populations, Rsb compares EHH of the same allele between different populations.

In this work, the iHS, xp-EHH and Rsb scores for each locus were calculated using the rehh package^24^ in R, and the candidate genomic regions under selection were obtained.

For each selection scan test, we defined the thresholds of 1% and 5% for the results to detect selected regions in the genome, with annotations referencing the sheep genome Ovis Oar_v4.0. We used UpSetR^25^ to analyze and visualize the cross-set relation of each gene set. Gene functional annotation was performed referencing the NCBI databases (http://www.ncbi.nlm.nih.gov/gene) and OMIM database (http://www.ncbi.nlm.nih.gov/omim).

### Bioinformatics analysis

STRING (http://string-db.org/), Gene Ontology database (GO, http://geneontology.org)^26^, Kyoto Encyclopedia of Genes and Genomes (KEGG, https://www.kegg.jp)^27^, Reactome^28^ and Human Phenotype Ontology (HP)^29^ were used for protein interaction, biological function and pathway analysis, respectively.

### RNA-Seq analysis

RNA was extracted from the tissues using the Qiagen RNeasy Plus Universal Kit. We prepared a kit sequencing library from KAPA Stranded RNA-Seq, and 100 bp double-ended reads were sequenced using an Illumina Next-Seq machine with a target of 50 million reads per sample. Bowtie2 was used to compare clean reads with the reference sequence. Then, RSEM was used to calculate the expression levels of genes and transcripts and establish the model of reads generation. Next, the maximum likelihood method was used to determine how to allocate reads to different transcripts. To achieve more accurate quantification, reads can be distinguished from positive and negative chains. In order to compare the expression levels between samples, it is necessary to standardize the gene expression levels. The standardized method used by RSEM is FPKM, and its calculation formula is as follows:

$$\begin{aligned} \mathbf {FPKM}= \frac{10^{6} C }{NL/10^{3}} \end{aligned}$$

Let FPKM(A) be the expression quantity of gene A, then C represents the number of fragments uniquely compared with gene A, N denotes the total number of fragments uniquely compared with the reference gene, and L is the number of bases in the encoding region of gene A. Using the FPKM method, the influence of gene length and sequencing amount difference in the gene expression can be eliminated, and the calculated gene expression amount can be directly used to compare gene differential expression among different samples.

### The analysis of gene selection sweep relation with RNA-Seq

The rows and columns of the FPKM constructed matrix were transposed to obtain a new matrix, in which each column corresponds to a sample. Then, we fitted this model through the least-squares method to calculate the t-statistic of the differential expression of each gene according to a standard method. The calculation formula is as follows:

$$\begin{aligned} t =\frac{(X^{T}X)^{-1}X^{T}Y[0]}{\sqrt{MSE\times (X^{T}X)^{-1}[0,0]}} \end{aligned}$$

where MSE is the mean square error of the fitted model:

$$\begin{aligned} MSE=\frac{1}{N}(Y-X(X^{T}X)^{-1}X^{T}Y)^{T}(Y-X(X^{T}X)^{-1}X^{T}Y) \end{aligned}$$

where N denotes the number of rows in x. The genes were sorted according to t-statistic results. The first 7% genes of each sample were defined as the specific expression gene set (SEGS) of the sample, and the result of the selective sweep of the genome was defined as the candidate gene set (CGS). Next, we took the intersection of the candidate gene set and the specific expression gene set of each sample and clustered them based on the data analysis of the ovaries in different periods while removing redundant results.

### qRT-PCR

A total of 15 genes were randomly selected from the genome selection signal results and transcriptome data analysis results. PRIMER5 was used to design quantitative primers. The primer sequences are shown in the Appendix Table S1, and the sheep 18s gene was used as an internal reference. We used the $2^{-\Delta \Delta Ct}$ analysis method to calculate the gene expression level^30^. For each sample, three biological replicates were used. By comparing the gene expression levels in different processed samples, the ratio of each gene expression was obtained.

## Results

### Population genetic structure and linkage disequilibrium

**Figure 1:**
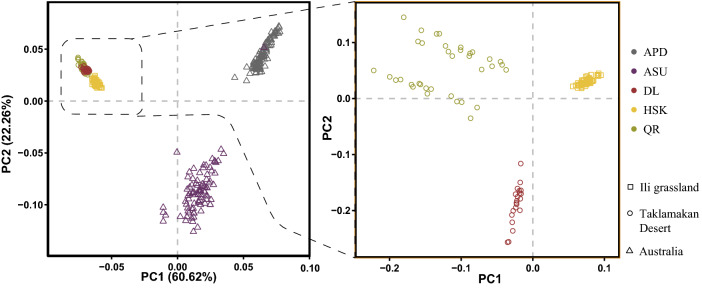
The PCA analysis chart of sheep breeds in three geographic regions, the X-axis represents PC1, and the Y-axis is PC2. The picture on the left shows the genetic distances of five sheep breeds, and that on the right shows the genetic distances of three sheep breeds in the Taklimakan Desert and the Yili Grassland.

After performing genome quality control, a total of 314 individuals and 45,298 SNPs were used for data analysis. As shown on the right side of Fig. 1, PCA divided the five breeds into three groups. In order to show the group classification more clearly, the Taklimakan desert environment sheep breeds and the Yili grassland sheep breeds were separately analyzed (Fig. 1right). The principal component (PC1) could separate the Duolang sheep, Qira Black sheep and Kazakh sheep.

**Figure 2:**
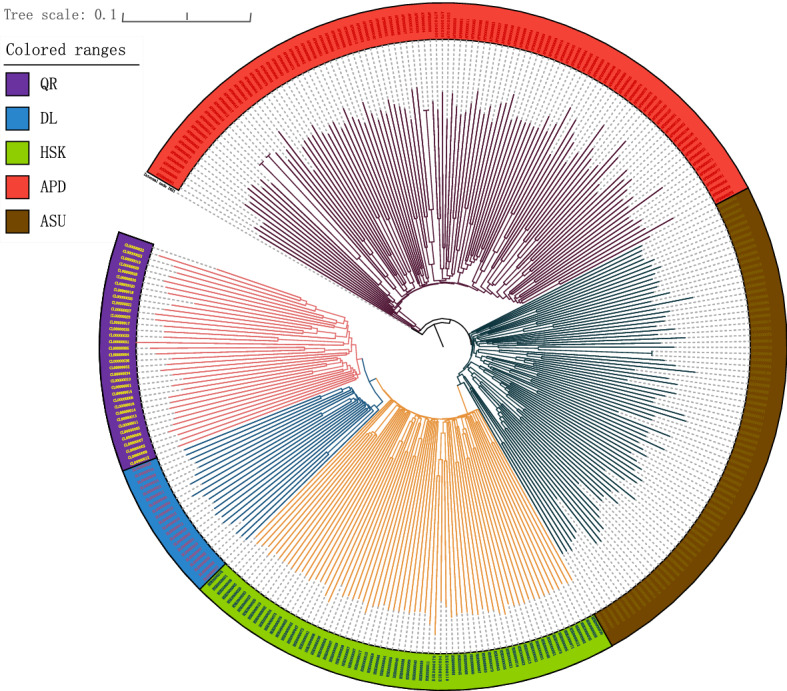
Evolutionary tree of five sheep breeds. Red represents APD, brown represents ASU, light green represents HSK, blue represents the DL and purple represents QR.

The results of adjacent evolutionary tree are shown in Fig. 2. Five sheep breeds were well differentiated according to the geographical regions. The Taklimakan Desert sheep breed is a branch, while the Yili grassland sheep breed represents a separate branch. The evolutionary tree shows that the Duolang sheep and Qira Black sheep are more closely related. When using the outer group APD as the root of the tree, the evolutionary tree shows that the Duolang sheep and Qira Black sheep evolved from the Kazakh sheep.

**Figure 3:**
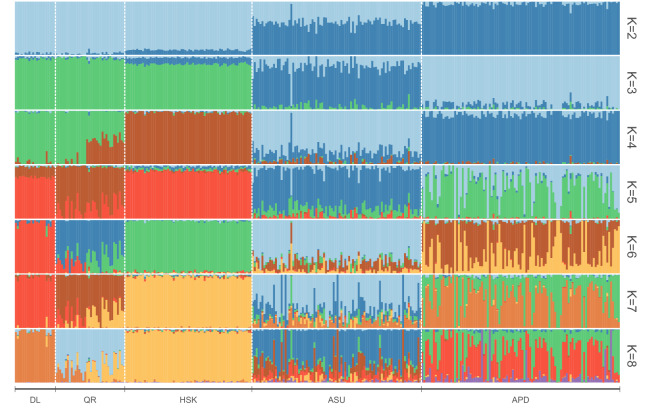
Admixture analysis of five sheep breeds. From left to right are DL, QR, HSK, ASU and APD.

In the admixture analysis, 28,568 of the 45,140 SNPs were deleted, and 16,572 SNPs were retained after LD pruning. The lowest CV error was detected when K = 8. QR, DL and HSK began to separate at K = 6, while APD and ASU began to separate at K = 3. When K = 5, QR, DL and HSK shared two main components. With respect to the size of the components, the evolution direction of the three was as follows: HSK$\rightarrow$DL$\rightarrow$QR. As K increased, QR, DL and HSK no longer produced obvious separation. The separation was mainly manifested in the two varieties of APD and ASU, indicating that APD and ASU may represent two artificially domesticated hybrids (Fig. 3).

**Figure 4:**
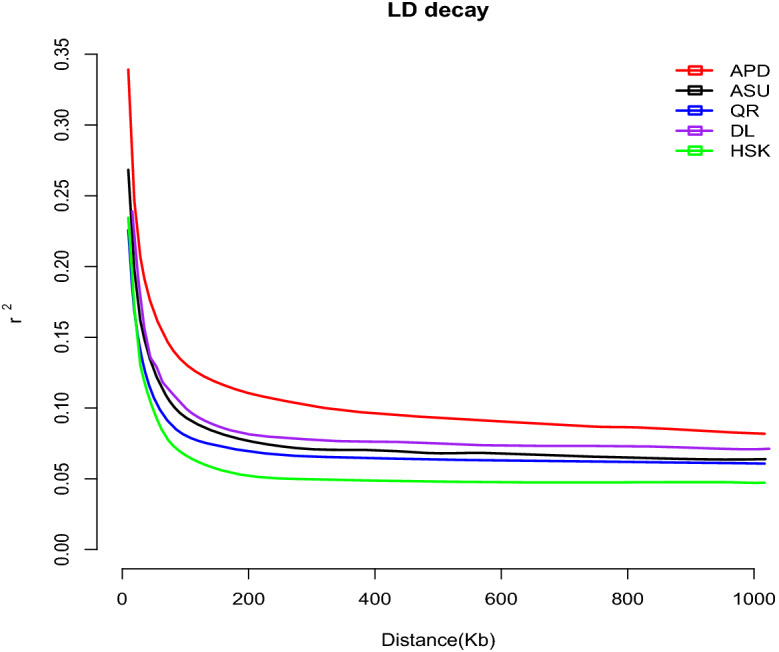
LD attenuation diagram of 5 sheep breeds. The X-axis represents the physical distance, while the Y-axis represents the linkage disequilibrium coefficient. Red represents APD, black represents ASU, blue represents QR, purple represents the DL, and cyan represents HSK.

The average LD coefficient $r^2$ of the three flock breeds QR, DL and HSK rapidly decayed within 10 kb. However, compared with the Taklimakan Desert sheep breeds, the Kazakh sheep from the northern foot of the Tianshan Mountains showed a faster decay. There was also a big difference with DL in the range of 50–300 kb (Fig. 4).

### Selection sweep, gene annotation and functional analysis

**Figure 5:**
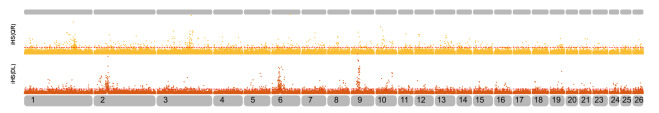
iHS Manhattan chart of QR and DL sheep breeds. The X-axis represents the chromosome, and the Y-axis represents the IHS value. The yellow part represents QR, and red represents DL. The red dashed line is the 1% threshold line.

**Figure 6:**
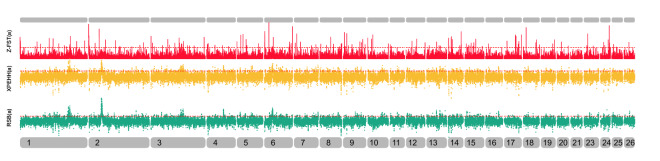
Manhattan chart of QR and DL versus HSK selective dissection analysis. The x-axis represents the chromosome, the green is the FST value standardized by the z-score, the orange is the xp-EHH value, and the black is the Rsb value.

**Figure 7:**
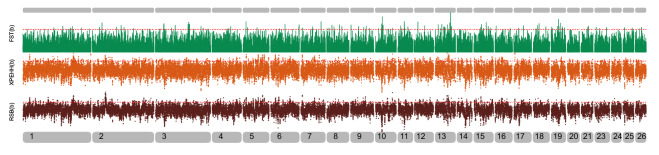
Manhattan chart of QR and DL vs. APD and ASU selective dissection analysis. The x-axis represents the chromosome, the green is the FST value standardized by the z-score, the orange is the xp-EHH value, and the black is the Rsb value.

The intersection and union relations of all selective scanning results of the iHS, FST, Rsb and xp-EHH methods are shown in Appendix Table S2. iHS was used to detect the selected areas of QR and DL (The results of QR are shown in Appendix Table S3 and those of DL are shown in Appendix Table S4, and their Manhattan diagrams are shown in Fig. 5). Common selected areas of QR and DL were mainly concentrated in OAR1, OAR3, OAR6, OAR9, OAR10, OAR19 and OAR26 at a threshold of 1%. Among the iHS screening genes, there were 526 intersection genes between QR and DL. These genes are related to the growth and development traits of organisms. For example, ACVR22, FADS2, FAR2, RARB, COL3A1, RERE, ITGAV, TEK and ADAMTS19 are related to animal cell development and metabolism^31–33^; KCNN3, FSHR, IGFBP7, CDC25B, CREB and FMR1 are related to early maternal follicular development and ovarian growth^34,35^; STX, KLHL1, BIN1, SCOT and KCNMB3 affect the ion channel function^36,37^. In the FST, Rsb, and xp-EHH tests, we used two paired comparisons: (a) QR and DL with HSK, (b) QR and DL with APD and ASU, and the results are shown in Fig. 6 and Fig. 7, respectively.

In the FST test, the following was obtained at a threshold of 1%: (a) 277 genes were detected in QR, DL and HSK, (b) 312 genes were detected in QR, DL, APD and ASU. The results of the FST test are shown in Appendix Tables S5 and S6. There were 63 gene intersections between (a) and (b), among which IHH, SORCS3, KCNMA1, TMTC1, EXT2 and HMGA2 are related to mammalian growth^38,39^; DSG4, RORA and LDLR are related to wool quality^40^; RNF157, CDH12, SIX2, SLC1A7, RXFP2 and R3HDM1 are related to adaptability in extreme environments^41–43^.

In the Rsb test, the following was obtained under the 1% threshold: (a) QR, DL and HSK detected 171 genes, (b) QR, DL, APD and ASU detected 175 genes. The Rsb test results are shown in Appendix Tables S7 and S8. There were 37 gene intersections between (a) and (b), among which CACNB2, CLDN23, CDC226, COMMD10, EIF2B5, NDUFAF2, OR10X1 and TENM2 are related to biological adaptability in extreme environments^44,45^; CLCN2 and SLC19A1 are related to ion transport^46^; ELOVL7, EP400 and MAPK8IP1 are involved in the sheep growth and development^47–49^.

**Figure 8:**
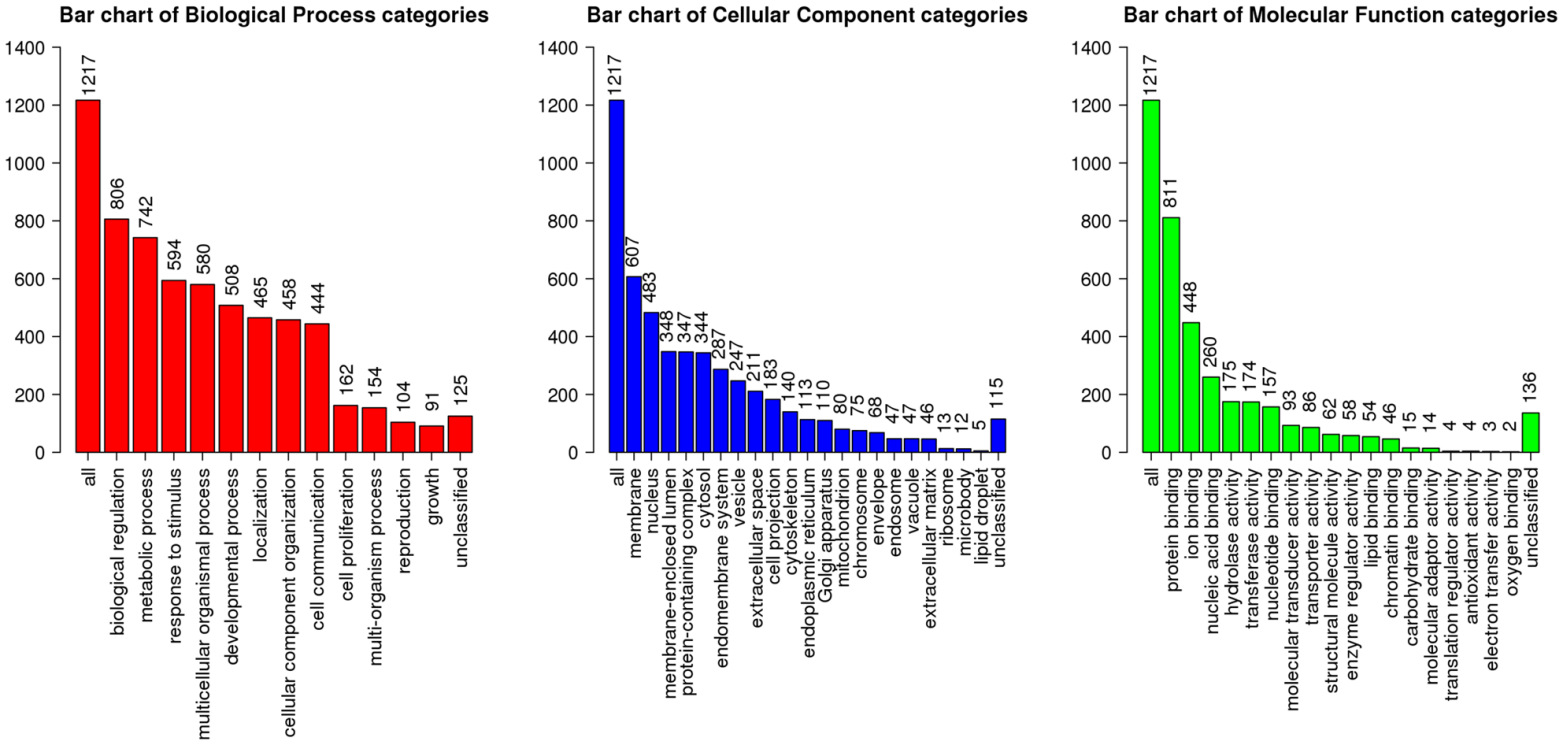
Selective sweep gene GO analysis at the top 1% threshold, from left to right are biological processes, cell composition and molecular functions.

In the xp-EHH test, the following was obtained under the 1% threshold: (a) QR and DL versus HSK detected 178 genes, (b) QR and DL versus APD and ASU detected 179 genes. The xp-EHH test results are shown in Appendix Table S9 and S10. There were 25 gene intersections between (a) and (b), among which KHDRBS2, LRP4, NEIL2 and SUV39H2 are related to biological adaptability in extreme environments^50,51^. Under the 5% threshold, 17 overlapping candidate genes were detected in IHS, FST, Rsb and xp-EHH tests, including ACVR2A, ATP11B, CCSER1, FXR1, GRID2, IGFBP7, KCNMB2, LRRC4C, MAT2B, NOX3, PAX1, RPL3, SPATA31E1, STPG2, TMTC2, USP25 and VRK2. GO enrichment analysis of significant biological processes for candidate genes under positive selective pressure revealed 51 Gene Ontology (GO) terms, shown in Appendix Table S11 and Fig. 8; the KEGG analysis resulted in 6 pathways, shown in Appendix Table S12. Furthermore, we obtained 50 Reactome pathways, listed in Appendix Table S13, and 2 Human Phenotype Ontology (HP) terms, as shown in Appendix Table S14.

### The analysis of gene selection sweep relation with RNA-Seq

**Figure 9:**
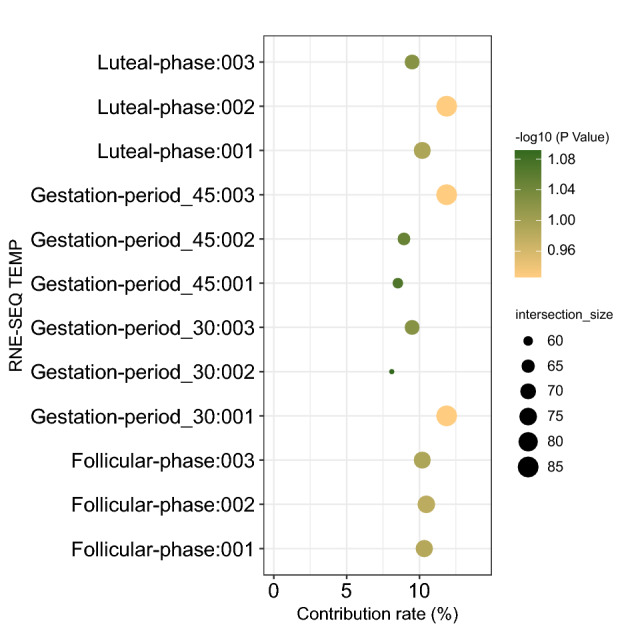
Candidate genes combined with selective sweeping and RNA-Seq analysis. The size of the scattered dots represents the number of genes, while the color represents the contribution of the specific gene set in each period to the total gene set.

We conducted correlation analysis between the results of RNA-Seq (shown in Appendix Table S15) and selection genome sweeping. As a result, we determined the top 7% of each sample’s specific expression gene set and its contribution (Fig. 9, Appendix Table S16). In the association analysis, the specific expression gene set at the follicular stage and the candidate gene set were found to share 17 intersection genes, of which NRG4 specifically binds to the EGF receptor ErbB4 (v-erb-b2 avian erythroblastic leukemia viral oncogene homolog 4). It exerts its physiological functions, such as stimulating cell proliferation, inhibiting cell apoptosis and improving the cell energy metabolism. For example, these functions include delaying female puberty^52^ and adult reproductive ability^53^. In this case, the overexpression of B4GALNT2 in the ovary leads to atypical glycosylation of inhibin (inhibin is an important hormone that regulates ovarian function), which promotes high fecundity in sheep^54^. In addition, previous studies have shown that this gene is related to follicular development and atresia^55^. EEPD1 maintains the genome stability during embryonic replication stress by promoting homologous recombination (HR) and inhibiting non-homologous end joining (NHEJ)^56^. The INTS9 complex promotes the recruitment of cytoplasmic dynein to NE and participates in RNA processing^57^. DNM2 plays a role in mediating endocytosis events that are critical to oocyte development; thus, it affects fertility^58^, MCAM is involved in melanin Invasion and tumor progression^59^ and mediates cell-endothelial cell interaction in the ovary^60^; this molecule also plays a role in the formation of new blood vessels during the formation of the corpus luteum in the human ovary^61^. As for OPN5, it belongs to an independent group separated from the other six groups in the phylogenetic tree of opsins, for which little information of absorption characteristics and molecular properties of the members is known^62^. PCDH17 can affect the sex differentiation of the gonadal through its expression level^63^. There were three shared genes between the specific expression genes in the luteal phase and the candidate genes set. The shared genes include FGF3, which is involved in the regulation of the corpus luteum (CL) growth and degradation^64^, CHKA, which encodes an enzyme involved in the synthesis of phosphatidylcholine^65^; phospholipid Acylcholine is the precursor of many physiologically important membrane molecules, including arachidonic acid and docosahexaenoic acid. These molecules are essential for male and female fertility^66^. The third shared gene is UQCRH, which is responsible for the electron transfer between cytochrome C and cytochrome C1, representing a part of the most critical step in the electron transfer cascade and being involved in the oxidative phosphorylation of mitochondria. In addition, UQCRH can protect the ovaries of animals from radiation-caused damage^67^.

The intersection set between the 30 days gestation specific gene set and the candidate gene set included 4 genes. Among them, F13A1 is related to the heat stress tolerance of pregnant ewes^68^, ARNTL indirectly affects the fertility of animals by affecting the circadian rhythm of the animal’s ability^69,70^. DNMBP has a role in membrane transport between the cell surface and the Golgi apparatus (through genetic similarity), and GLIS3 affects the development of organs, such as the fetal pancreas or thyroid^71–73^.

A total of 5 genes resulted in the intersection set between the 45 days gestation specific expression gene set and the candidate gene set. Among them, HTR1A promotes the fetal-maternal tolerance of the endometrium by mediating immunosuppression^74^, AS3MT (Arsenic (+3 oxidation state) methyltransferase) is a gene that depends on the sex of the fetus and represents a key enzyme in the metabolism of inorganic arsenic (iAs)^75^, GNAQ is a candidate mediator of melanopsin signal transduction, which can affect the fetal hyaline and retinal vascular development^76^, CCDC178 may lead to fetal atrial septal defect and cardiovascular development abnormalities if in abnormal state^77^, and CNGA1 participates in the coding loop cyclic nucleotide-gated channel (CNG channel), affecting pregnancy in animals^78^.

### qRT-PCR

**Figure 10:**
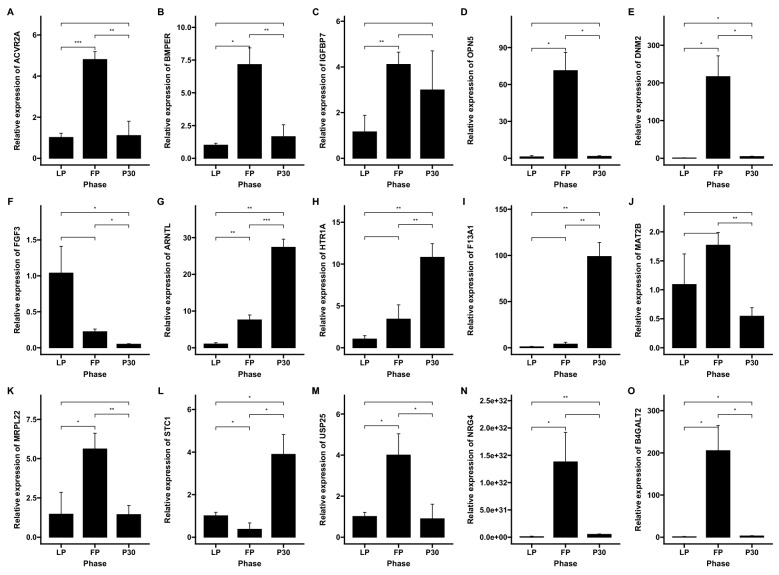
qRT-PCR analysis of 15 genes in the ovarian follicular phase, luteal phase and gestation period of Qira black sheep. The x-axis represents three different periods of organization, while the y-axis represents the level of gene expression. The candidate genes were selectively cleaned to detect the expression of sheep ovaries in three different periods.

A total of 15 genes were randomly selected for qRT-PCR verification to verify their expression in ovarian tissues at three different periods. The results are shown in Fig. 10. It could be seen that ACVR2A was up-regulated during the follicular phase (FP) and down-regulated during the luteal phase (LP) and pregnancy (P30). The difference between FP versus LP and P30 were highly significant. BMPER and MRPL22 were up-regulated during FP and down-regulated during the LP and P30. The difference between FP versus LP was significant, and that between FP versus P30 was very significant. IGFBP7 was up-regulated in FP and down-regulated in the LP. The difference between LP versus FP was highly significant. OPN5 was up-regulated during FP and down-regulated during LP and P30. There was a significant difference between LP versus FP and a significant difference between LP versus P30. B4GALT2 and DNM2 were up-regulated during FP and down-regulated during LP and P30. There was a significant difference between LP and FP as well as between LP and P30, and a significant difference between FP and P30. NRG4 was up-regulated during FP and down-regulated during LP and P30. The difference between LP versus FP was significant, and that between LP versus P30 was very significant. FGF3 was up-regulated during LP and down-regulated during FP and P30. There was a significant difference between FP versus LP and P30, and also between LP versus P30. USP25 was up-regulated during FP and down-regulated during LP and P30. FP versus LP and P30 were significantly different. ARNTL, HTR1A and F13A1 were up-regulated during P30 and down-regulated during LP and FP. P30 versus FP and LP were significantly different. STC1 was up-regulated during P30, and down-regulated during LP and FP. P30 versus FP and LP were significantly different. MAT2B was up-regulated during FP and down-regulated during P30. The difference between FP versus P30 was extremely significant.

## Discussion

### Genetic diversity and population structure

PCA analysis showed the selection of sheep breeds in the Taklimakan Desert to be different from other agro-climatic breeds. The resulting cluster relationship of the five sheep breeds was closely related to their geographical distribution. The further the geographical relationship, the higher the discrimination through PCA. The evolutionary tree revealed DL and QR to be evolved from HSK. These findings are consistent with the study of Yang et al.^11^. Admixture analysis revealed the degree of genomic mixing of the 5 sheep breeds. The ancestral components of K = 2 to K = 4 were related to the geographical origin. At K = 2, a small amount of mixing was detected between Australian and Chinese breeds, which might be because part of the living environment of Australia and Xinjiang sheep in China is the same. For K = 5, the three Xinjiang varieties were roughly divided into two parts (DL and HSK versus QR), and they were not mixed with Australian varieties. This indicates that HSK is more closely related to DL and farther from QR. When K = 8, the three varieties of Xinjiang could be separated. When there was still a small amount of mixing, it was mainly manifested in QR and DL. This shows that QR and DL co-evolved, and there was also a phenomenon of gene exchange, but QR has evolved further. These results are consistent with the conclusions of PCA and adjacent evolutionary trees.

### Selection sweep methods

Different tools of biological analysis have different results, but no one method can completely detect all the biological results in a genome-wide selective sweep analysis^19^. We improved the accuracy of our data using a variety of genomic selection methods. Selected sites of DL and QR varieties were scanned by iHS. The methods of FST, xp-EHH and Rsb were used to scan the selected difference sites of (a) QR and DL versus HSK, and (b) QR and DL versus APD and ASU. The results revealed genetic evidence of the adaptation of sheep breeds in the Taklimakan Desert to local environments. In the results of genome selective cleaning, 1,279 genes were collected under the threshold of 1% (Appendix Table S17), while there are 17 overlapping genes under the threshold of 5%.

### Adaptive mechanism of the desert environment

The environment adaptability differs between the Taklimakan Desert sheep breeds and those of other areas. Taklimakan Desert indigenous sheep breeds can adapt to extreme conditions, such as high salinity, drought and high ultraviolet rays. Using four complementary genomic selective sweeps, 1279 genes were obtained under the 1% threshold, and the biological enrichment results of these genes showed the genetic evidence and physiological mechanism of the adaptation of native sheep to the desert environment.

In terms of adaptability, $Ca^{2+}$ activated $K^{+}$ channels (R-HSA-1296052) promote cytoplasmic $Ca^{2+}$ accumulation. Then, they reduce $K^{+}$ elimination and cytoplasmic $Na^{+}$ accumulation^28^. cGMP affects (R-HSA-418457) by affecting phosphodiesterase (PDE) and cGMP-dependent protein kinase (cGK, Protein Kinase G or PKG); it can regulate vasodilation, platelet aggregation and neurotransmission, as physiological functions^28^. In addition, among the target genes of the association analysis with the transcriptome, F13A1 in the 30 days gestation component is related to the heat stress tolerance of pregnant ewes^68^.

In terms of immunity, 5 genes (DNTT, FEN1, POLL, PRKDC and XRCC4) participate in the non-homologous end-joining pathway. XRCC4 and DNTT are involved in repairing the DSB repair caused by exogenous or endogenous DNA damage as well as the DSB repair produced in the process of V(D)J recombination^79^. Eight genes (ABCC1, ABCC2, ABCD4, ABCC5, ABCC6, ABCC11, ABCC12 and ABCB10) are involved in the ABC transporters pathway. These proteins transport a variety of substrates across the membrane, and they can cause cystic fibrosis, neurological diseases, retinal degeneration, cholesterol and bile transport defects, anemia and drug reactions when mutated^80^. MAPK (R-HSA-5683057) and PI3K/Akt (hsa04151) activate the macrophages induced by ASKP-1, thereby enhancing immune regulation^28^. Under a high radiation environment, the SUMOylation of DNA damage response and repair proteins (R-HSA-3108214) pathway is involved in DNA damage response and repair^28^.

The results of the biological enrichment of candidate genes using the Human Phenotype Ontology database were mainly related to vision. The 10 genes dominate eye development, pigment changes and light perception. Among them, MAFB regulates the lens development^81^. As for PTEN, it affects the cell size and inhibits the cell cycle process of early mitosis, through inactivation and overexpression, thereby promoting eye growth and development^82^. MITF plays a central role in the regulation of neuroepithelial domains and also the differentiation of retinal pigment epithelium, promoting the development of the normal retina and pigment epithelium^83^. Regarding EDN3, it plays a role in the development of ocular angiogenesis in the early postnatal period^84^. Rapid and accurate eye development represents the basis of vision development. These genes regulate eye development at different stages and positions. They are candidate genes for vision-related formation in indigenous sheep after long-term breeding. PNPLA6 encodes a protein that plays an important role in photoreceptor survival^85^. SNAI2 can regulate the extracellular matrix (ECM) protein of trabecular meshwork (HTM) cells, which in turn play a key role in aqueous humor circulation^86^. These genes promote the eye development of indigenous sheep and the development of their observation ability and agility. Eye pigments are produced by pigment cells, and pigments are directly related to vision and radiation resistance. The Taklimakan Desert sheep breed has long lived in a high ultraviolet environment, and the eyes and other organs have correspondingly adapted. For example, sun exposure may cause the development of melanoma, and GNAQ mutations can affect uveal melanoma (UM)^87,88^. Some genes determine the eye color and protect melanocytes from radiation, such as ACTB, KITK and ITLG^89,90^. In the genome-wide and transcriptome association analysis, the follicular phase-specific and significantly expressed gene OPN5 (neuropsin) is a bistable pigment that is sensitive to ultraviolet light^62^, which is related to the adaptability of the Taklima Desert breeds to the high ultraviolet environment. Compared with other domesticated sheep breeds, indigenous sheep breed of the Taklimakan Desert have better vision and more alertness, and these characteristics are related their foraging and avoiding of natural enemies in extreme desertification environments.

### Genetic mechanism of perennial estrus and reproduction

The animal estrus cycle is the alternate process of FP and the LP, which affects follicular development. The formation and degeneration of the corpus luteum results from the joint influence of the genes and environment. Sheep breeds underwent natural and artificial selection in the environment of the Taklimakan Desert for a long time, and have developed stable reproduction and perennial estrus.

By combining whole-genome and RNA-Seq, a series of genes that are related to development and reproduction were found, mainly in the following two aspects.

FGF3 regulates the growth and degeneration of the corpus luteum^64^, PCDH17 is involved in the stabilization of sex differentiation of the gonadal^63^, and MCAM mediates the cell-endothelial cell interaction in the ovary^59^. ARNTL^69,70^, CHKA^65^ and NRG4^53^ guarantee the balance and supply of hormones and reproductive factors during sheep reproduction, thus improving the maternal reproductive ability. B4GALNT2^54^ and DNM2^58^ affect the follicular development and related physiological processes, hence promoting high animal production. Indigenous sheep use these genes to increase the reproductive rate and achieve multiple birth rates, directly or indirectly and in different ways, which is necessary for the continuation of the population in harsh environments. F13A1 improves heat stress tolerance of ewes^68^, while UQCRH protects the organisms from radiation-induced ovarian damage^67^, which is essential for sheep living in desert environments. As for CCDC178 and CNGA1, they are involved in immune repair^77,78^, while GLIS3^71–73^ and GNAQ^76^ are involved in stable fetal organ development. EEPD1^56^ plays a role in maintaining the stability of embryonic genome replication. HTR1A^74^ mediates immunosuppression to improve fetal-maternal tolerance, which is essential to reduce the miscarriage rate. Indigenous sheep have evolved a more unique and stable reproductive system to ensure the stability of pregnancy in the harsh desert environment.

The Reactome results of the enrichment analysis included the TFAP2 (AP-2) family, which regulates the transcription of growth factors and their receptors (R-HSA-8866910) pathway to ensure stable cell differentiation and proliferation^91^. The quantitative analysis of randomly selected genes showed the STC1 gene to be highly expressed in the ovary of sheep, such that the expression level is higher in the follicular phase, significantly decreases after entering the luteal phase, and reaches the highest level at 30 days of pregnancy. STC1 is a glycoprotein hormone that appears to have autocrine/paracrine effects in several mammalian tissues^92^. It is involved in a variety of biological processes, such as apoptosis^93,94^, inflammation^95^ and reproduction^96^. Previous studies have shown STC1 to be highly expressed in the ovaries of humans and mice^97,98^. Harminder found the STC1 protein to mainly exist in the egg membrane and oocytes. Although it is expressed in the corpus luteum, the expression level is low and regulated by the luteinizing hormone (LH)^92^.

STC1 in the human follicular fluid can play a certain regulatory role when it is non-covalently bound to the pregnancy-associated plasma protein (PAPP-A), and it can inhibit the biosynthesis of progesterone downstream of adenylate cyclase^99^. Treatment of granulosa cells with STC1 can reduce the stimulation of gonadotropins for progesterone production and LH receptor formation, without affecting the secretion of estradiol by gonadotropins^100^; thus further indicates the potential role of STC1 as a luteinizing inhibitor. STC1 co-treatment significantly inhibits the transcription of the rate-limiting enzyme CYP11A of progesterone biosynthesis, induced by FSH^101–103^. During pregnancy, STC1 levels continue to rise, and on the 10th day of pregnancy, it reaches 15 times the level of normal cycle mice, and STC1 can then be detected in the serum^92^. These reports on STC1 in the estrus cycle and pregnancy indicate that STC1 plays an important role in mammalian ovarian physiology and reproduction. This raises the need for more studies on its specific functions.

IGFBP7 is abundantly expressed in the ovaries, and its expression significantly rises after entering LP from FP and decreases after entering the pregnancy period. This gene is involved in the formation of vascular endothelial growth factor (VEGFA) and LH. VEGFA and LH stimulate the formation and proliferation of LEC (microvascular endothelial cells), which indicates that the secretion of IGFBP7 in the corpus luteum may inhibit VEGFA, thus affecting early CL angiogenesis^104^. This gene is expressed at a high level throughout the growth of antral follicles of buffalo and may thus have an impact on fertility by regulating the growth of follicles^105^.

Finally, the MRPL22 gene is abundantly expressed in LP. Its expression significantly increases after entering the luteal phase from the ovarian stage and significantly decreases after entering the pregnancy period. It was revealed by related research on the in-depth characterization of the mouse embryo mutant MRPL22 that the absence of MRPL22 led to severe developmental delay and the inability to initiate gastrulation on the 7.5th day of the embryo. Besides, it was shown by related metabolic analysis that the mitochondrial function of MRPL22 knockout embryos was impaired, which resulted in a significant decrease in ATP production l^106^.

**Figure 11:**
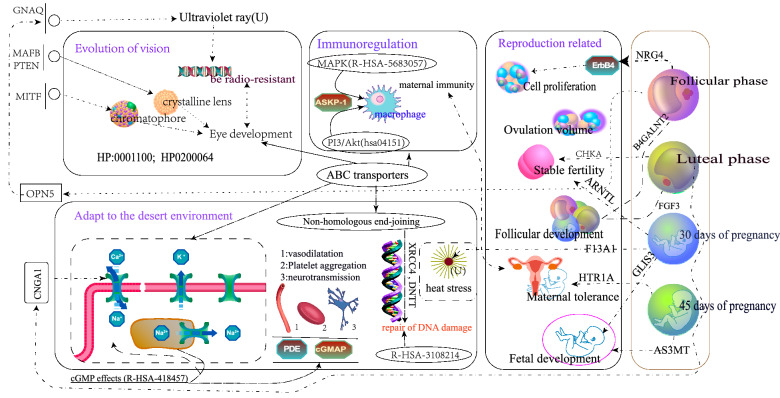
Environmental adaptability and perennial estrus regulatory network of the Taklimakan Desert sheep breeds.

In a word, the stable production of sheep requires accurate and rapid development, precise immune control and tolerance to the environment. To achieve high sheep production, follicular quality and high ovulation rate are essential. In the Taklimakan Desert, native sheep breeds have formed a unique reproduction and immune control mechanism through natural and artificial selection. This physiological mechanism allows them to survive in harsh environments. Their adaptation to the desert environment is a complex and continuous process. Figure 11 briefly describes its physiological mechanism and relationship from the aspects of reproduction, drought, salt and alkali tolerance as well as vision development.

### New Views on breeding of tolerance sheep breeds

The main focus in sheep breeding is on economic benefits, e.g., cultivating new varieties with high-quality meat and wool. Genetic drift and selection represent the main two sources of genetic evolution. Random genetic drift will cause losing the genetic diversity and irreversible consequences, such that the reduced genetic diversity will limit the ability of the introduced population to evolve in its new or changing environment. To avoid this, we propose two solutions: First, adopting supportive breeding, which means bringing some individuals of wild populations into captivity for breeding, and performing artificial selection in the offspring. While this program aims to improve the environmental adaptability of domesticated populations, it has become more difficult to be implemented with the shrinking of the wild sheep population. Second, cultivating conservation breeding for economic crops. This strategy protects the species diversity in extreme environments and preserves and domesticates populations in extreme environments. These proposed measures can enhance the adaptability of species in the new environment, such that they can deal with unpredictable climate changes in the future.

## Conclusions

This work used four complementary genomic selection signal analyses to reveal the genetic mechanism of stress resistance of indigenous sheep breeds in the Taklimakan Desert environment. We successfully constructed selection signal maps of sheep breeds in three different geographical and ecological environments. In addition, we elaborated on the physiological system of sheep breeds in the Taklimakan Desert, including immunity, vision degeneration, high reproduction rate and water reabsorption, and we constructed the prediction mechanism map of desert adaptability. Through the combined analysis of genomic selection signal and transcriptome, we analyzed the genetic mechanism of perennial estrus and stable reproduction of indigenous sheep in the Taklimakan Desert environment, and the related genes of B4GALNT2, FGF3 and F13A1 were mainly involved. These results help to understand the genetic mechanism behind the adaptability of sheep breeds in desert environment at the molecular level and provide new ideas for breeding tolerant sheep breeds in extreme environment.

## Supplementary Information

Supplementary Tables.

Supplementary Information.

Supplementary Legends.
