# Supplementary material for: Analysis on the desert adaptability of indigenous sheep in the southern edge of Taklimakan Desert
Source: Sci Rep. 2022 Jul 18;12:12264. doi: 10.1038/s41598-022-15986-x (PMC9293982; doi:10.1038/s41598-022-15986-x)
Supplement: Supplementary file 2 — Supplementary Information. [file 41598_2022_15986_MOESM2_ESM.pdf]

Appendix

Fluorescence quantification

Experimental background

Quantitative real-time PCR (Quantitative real-time PCR) is an accurate and rapid nucleic acid quantitative analysis technology developed in the 1990s. By introducing a fluorescent chemical substance into the PCR reaction system, the fluorescent signal of each cycle product in the PCR amplification reaction is detected in real-time, and a fluorescent amplification curve is obtained. This experiment is used to quantitatively analyze the initial template. Relative quantification refers to the change in the amount of the target sequence in the sample to be tested relative to another control sample, comparing the level of gene expression changes in two or more samples with different treatments, and the result is a ratio. In this process, certain internal reference genes should be selected to remove possible differences in RNA yield, quality, and reverse transcription efficiency of different samples, and perform correction and standardization.

Experimental results

The primer information of the 16 genes to be quantified in the experiment

For primer, information see attached table S17

Amplification curve and melting curve

Amplification plot: In the PCR process, take the cycle number as the abscissa and the real-time fluorescence intensity during the reaction as the ordinate as the standard curve. The amplification curve shows two phases, the exponential growth phase and the non-exponential plateau phase that follows. In the exponential growth phase, the amount of PCR product in each cycle approximately doubles. However, as the reaction progresses, the components of the reaction system are consumed, and one or more of the components restrict the reaction. At this time, the product growth rate slows down, and the reaction enters a plateau.

Amplification curve result diagram of 15 genes:

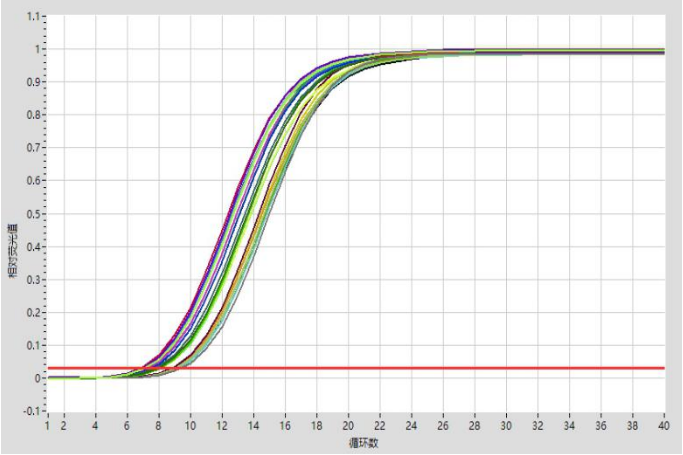

Figure 1- 1 18S

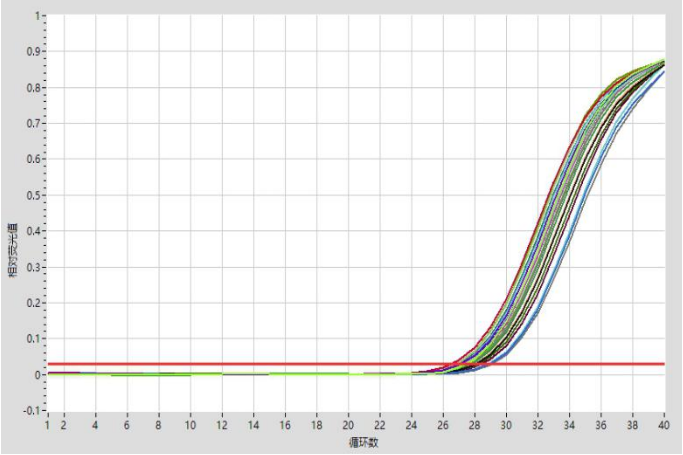

Figure 1- 2 ACVR2A

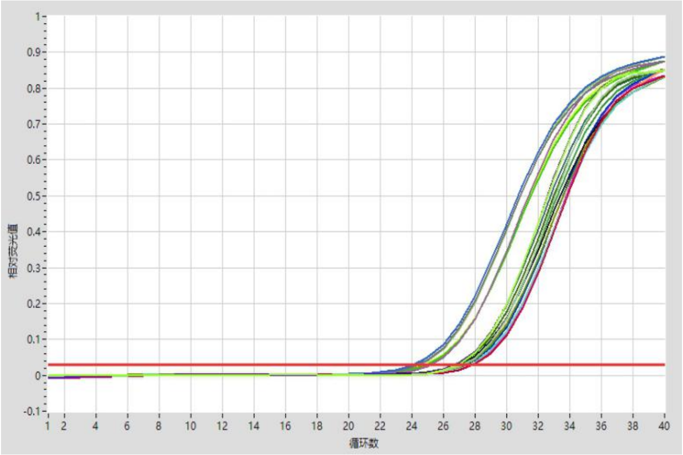

Figure 1- 3 B4GALT2

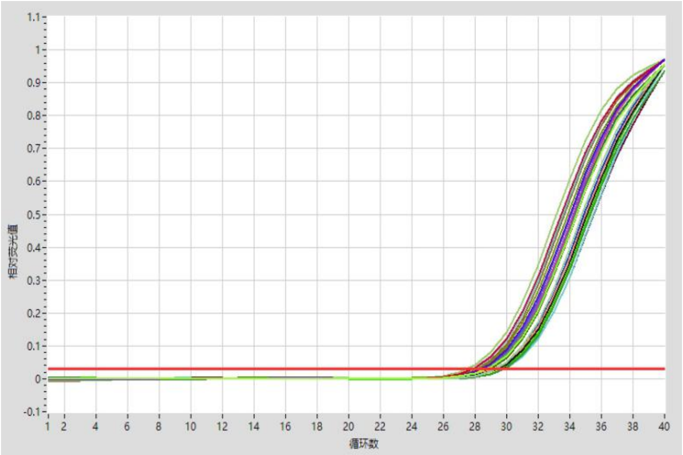

Figure 1- 4 BMPER

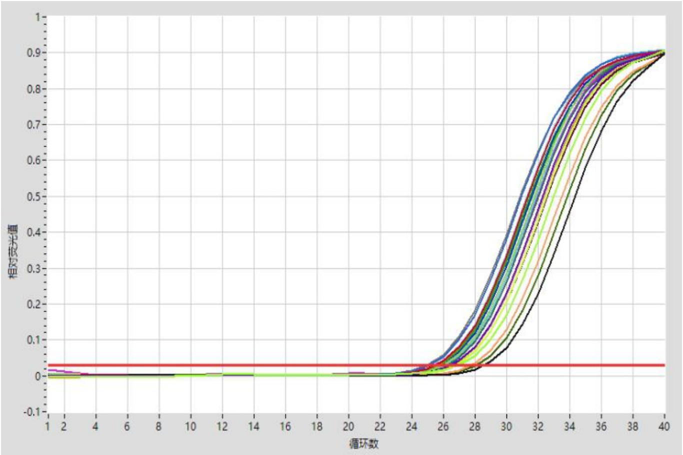

Figure 1- 5 DNM2

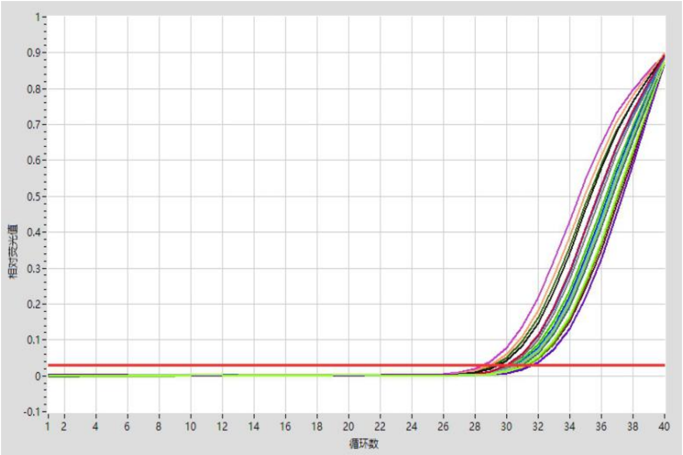

Figure 1- 6 FGF3

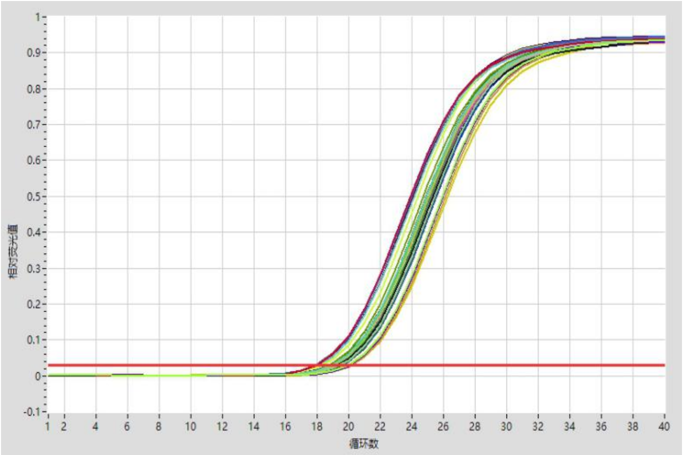

Figure 1- 7 IGBP7

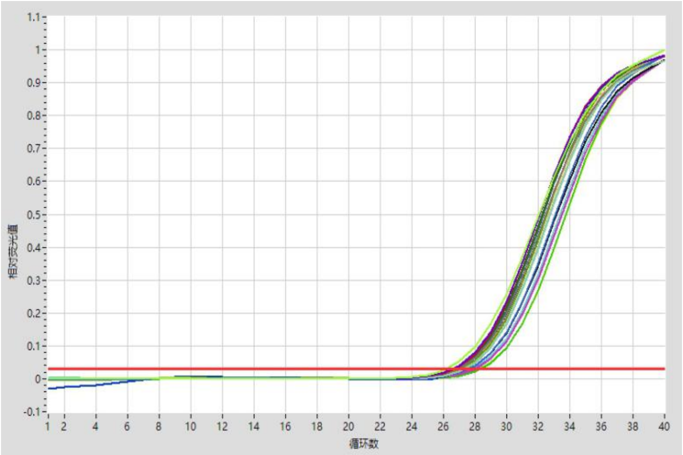

Figure 1- 8 MAT2B

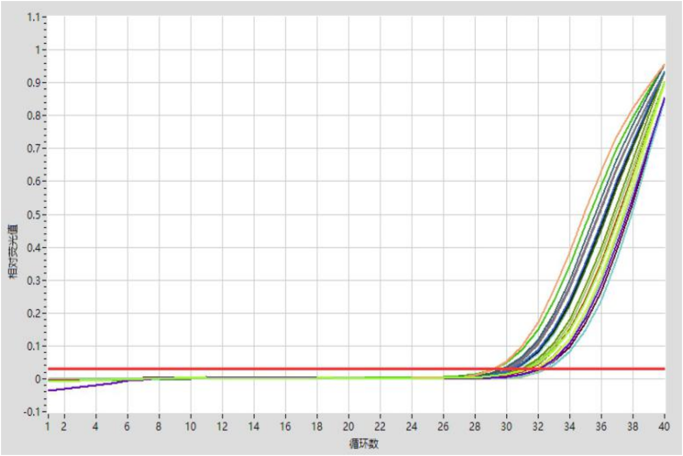

Figure 1- 9 MRPL22

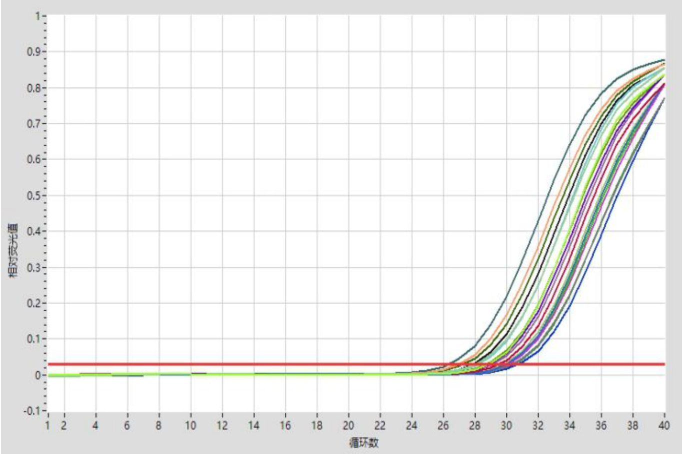

Figure 1- 10 NRG4

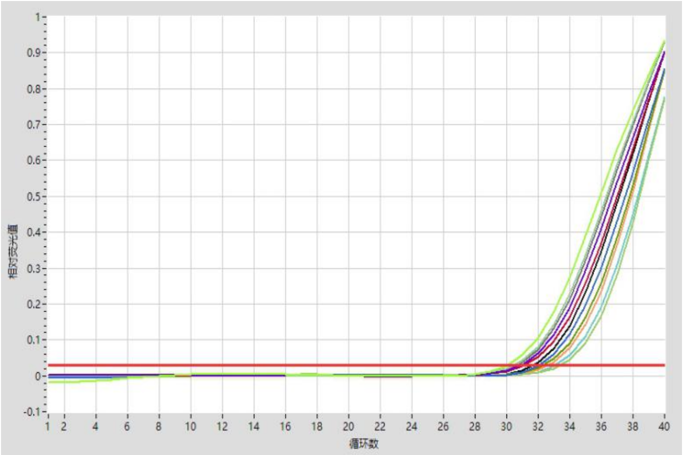

Figure 1- 11 OPN5

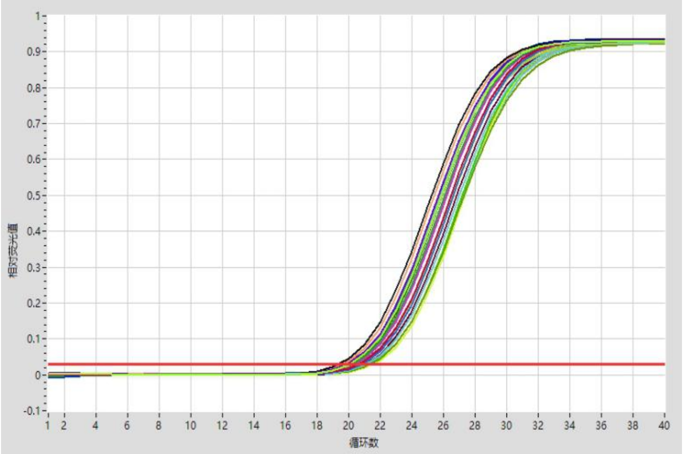

Figure 1- 12 RPL3

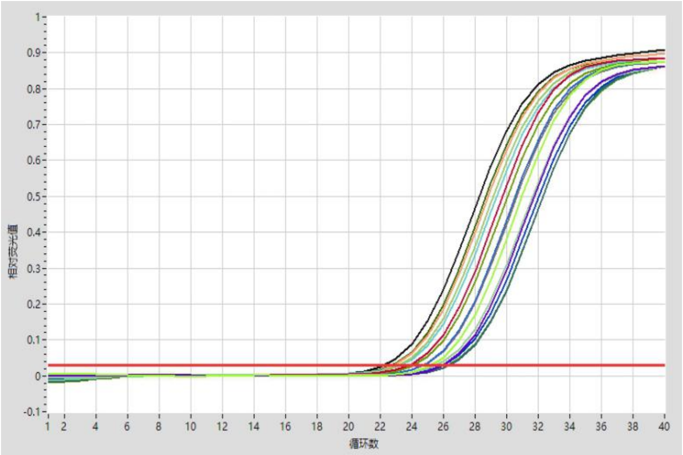

Figure 1- 13 STC1

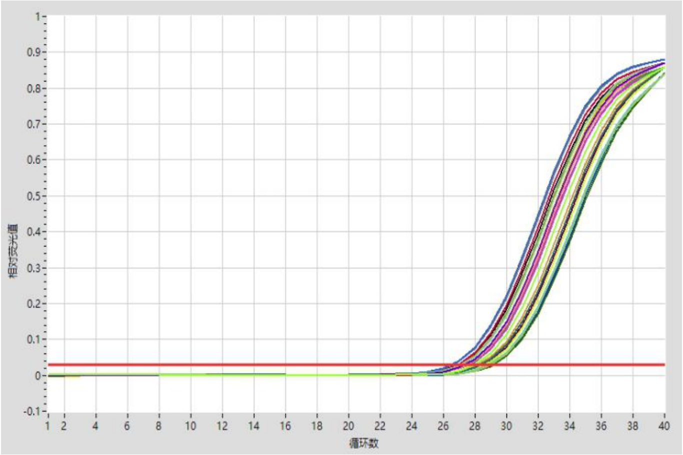

Figure 1- 14 USP25

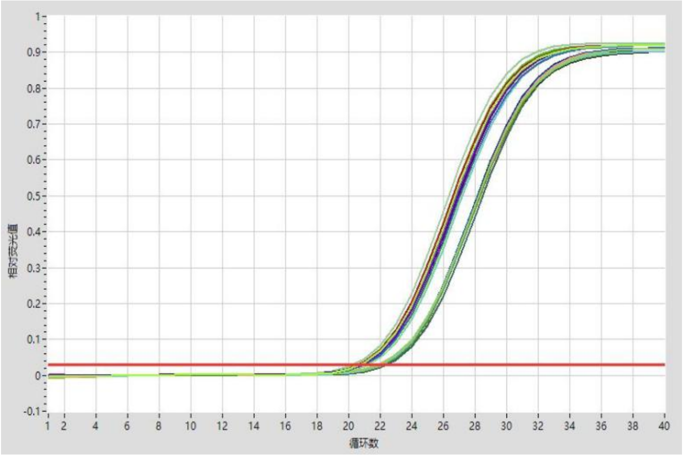

Figure 1- 15 HTR1A

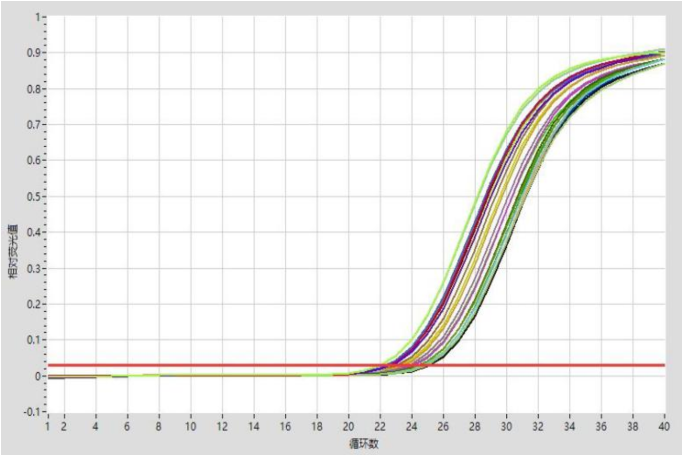

Figure 1- 16 ARNTL

Melt curve: The curve of the degree of degradation of the double helix structure of DNA with increasing temperature. The temperature at which half of the total DNA double helix structure is degraded is called the melting temperature (Tm), Different DNA sequences have different Tm values. The higher the G-C content in DNA, the higher the Tm value, which is directly proportional. If the melting curve has multiple peaks, it proves that the reaction is non-specific or the presence of dimers.

Results of melting curves of 15 genes

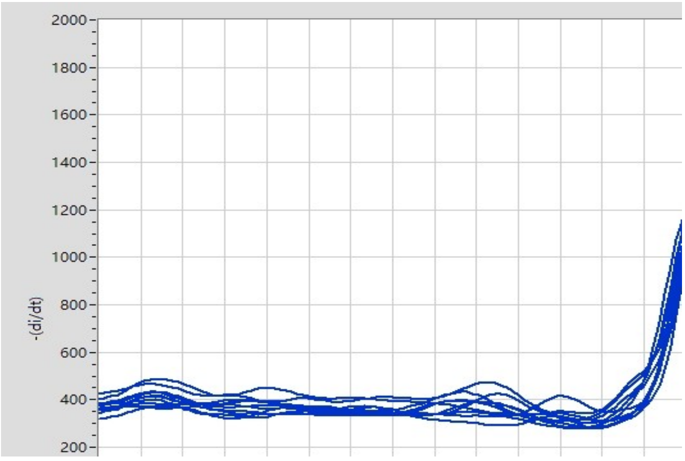

Figure 2- 1 18S

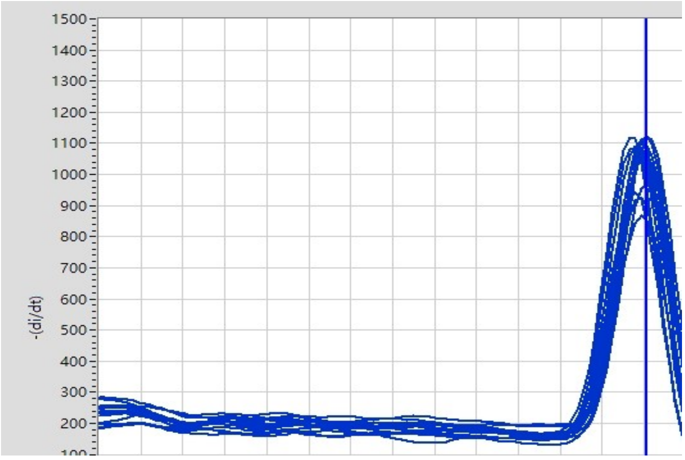

Figure 2- 2 ACVR2A

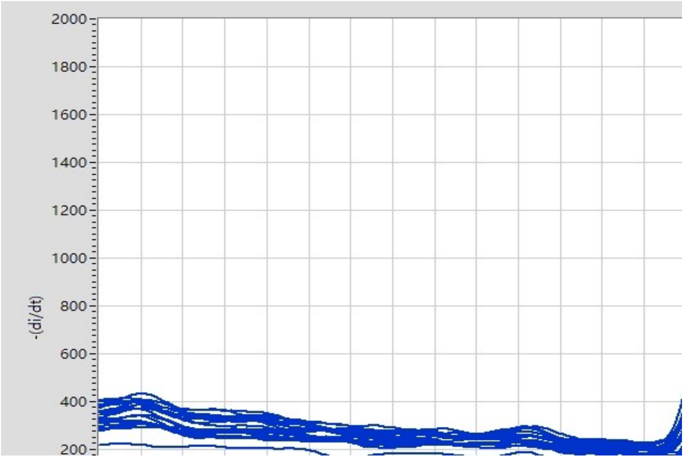

Figure 2- 3 BMPER

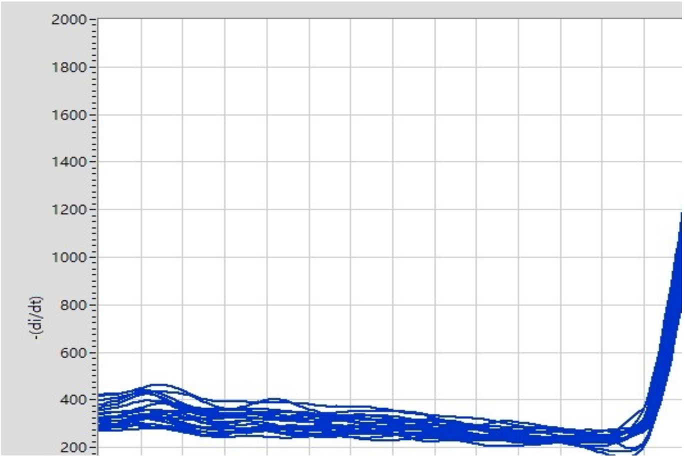

Figure 2- 4 IGFBP7

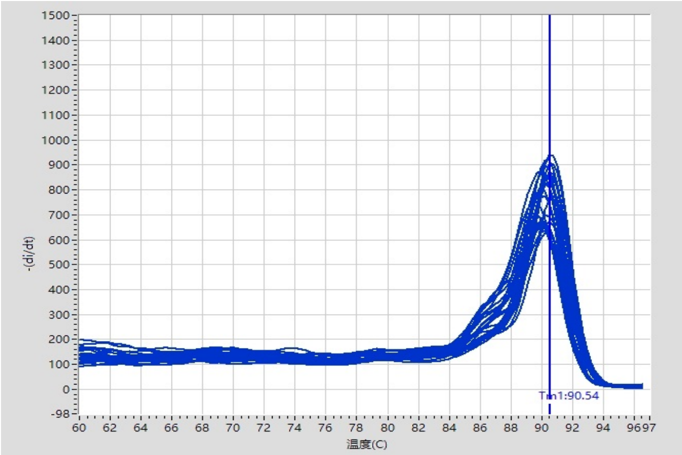

Figure 2- 5 OPN5

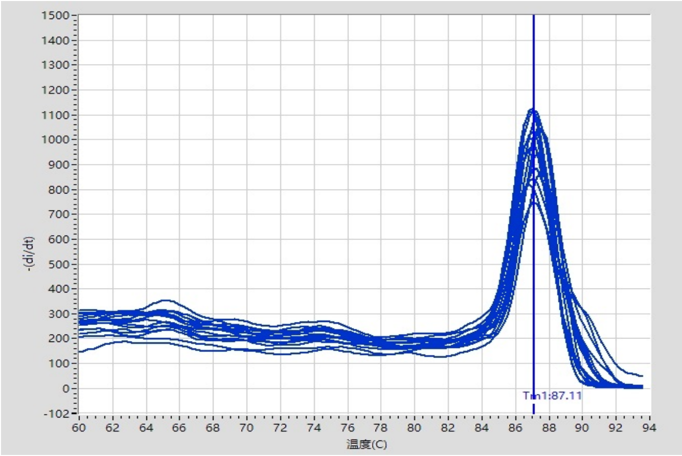

Figure 2- 6 DNM2

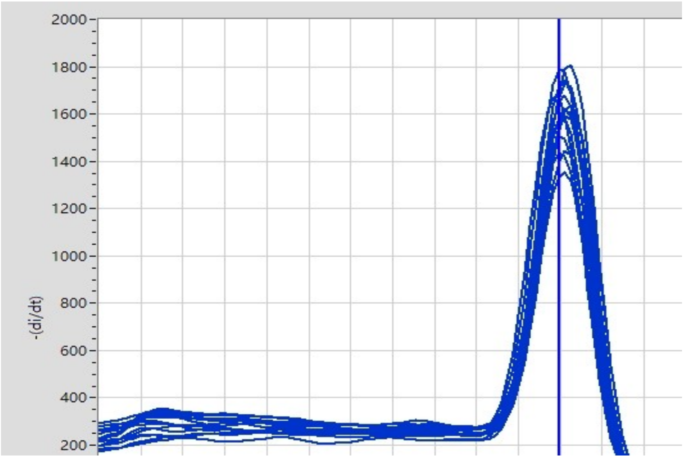

Figure 2- 7 FGF3

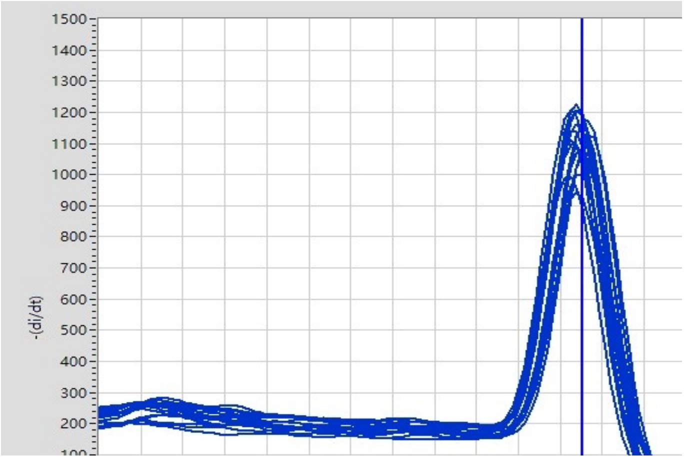

Figure 2- 8 ARNTL

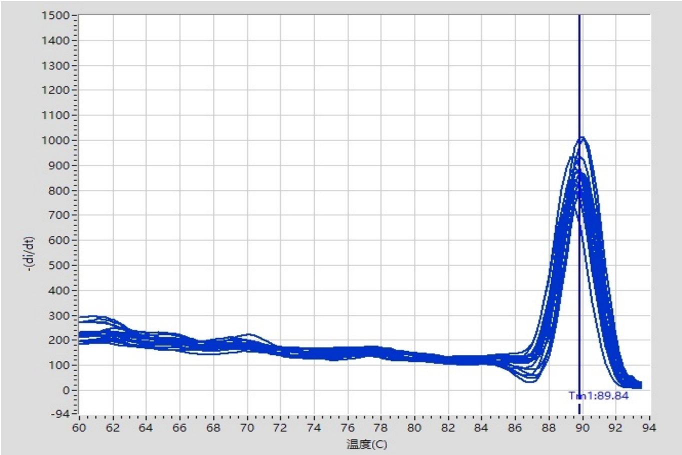

Figure 2- 9 HTR1A

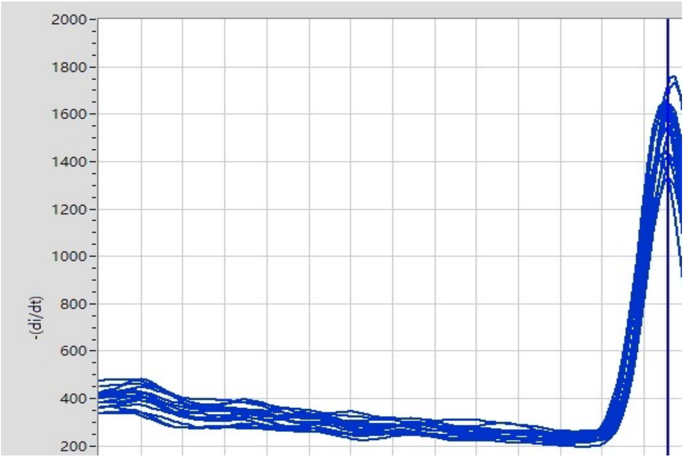

Figure 2- 10 F13A1

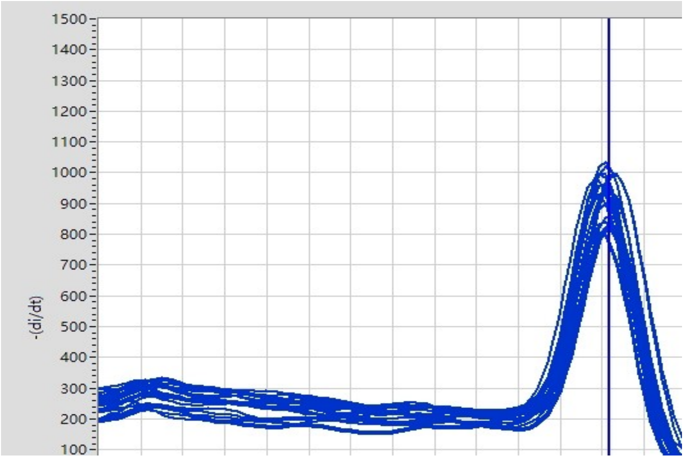

Figure 2- 11 MAT2B

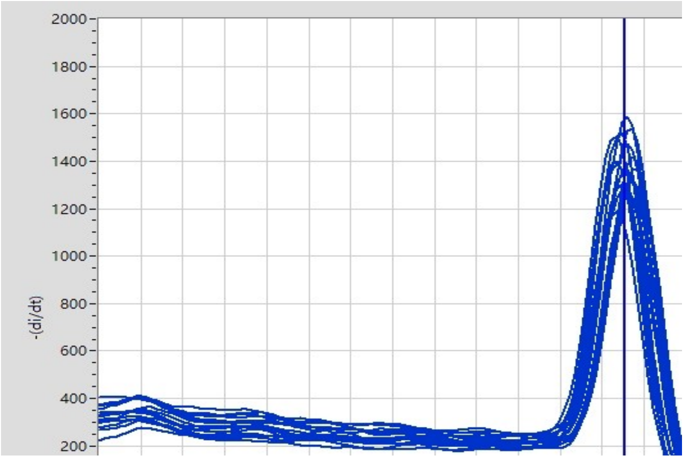

Figure 2- 12 MRPL22

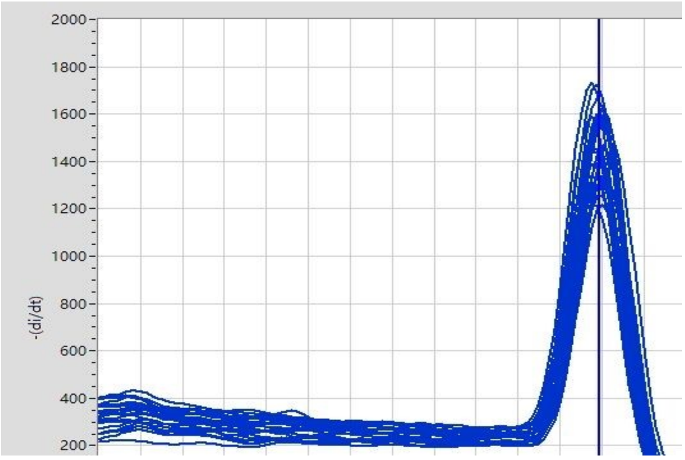

Figure 2- 13 STC1

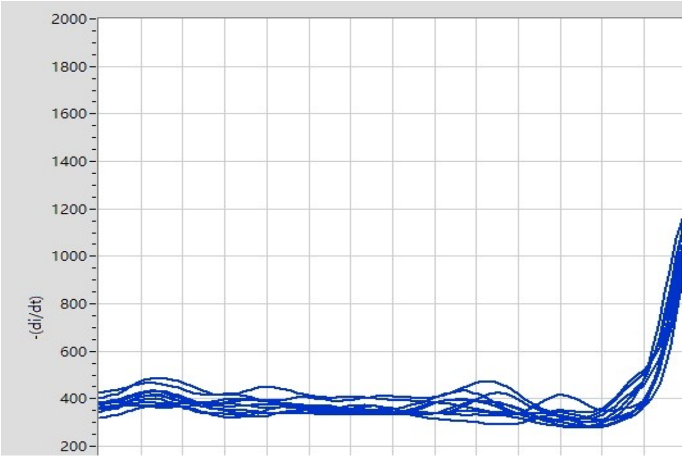

Figure 2- 14 USP25

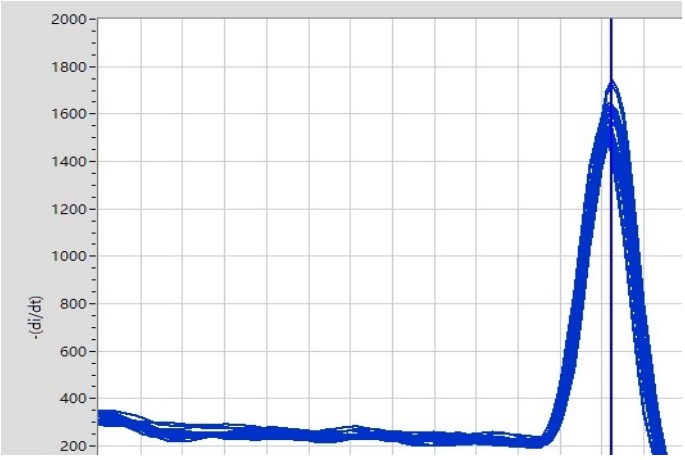

Figure 2- 15 NRG4

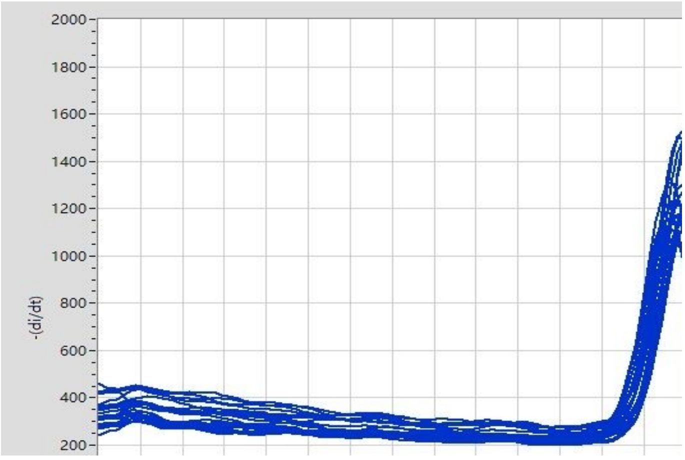

Figure 2- 16 B4GALT2

Experimental method

Experimental Materials

Three ovaries of Duolang ewes in the follicular phase, luteal phase, and 30 days of gestation were selected. They were cut with sterile surgical scissors and placed in a cryopreservation tube. They were quickly stored in liquid nitrogen and transferred to a -80°C refrigerator. In the spare. The experimental materials were provided by the Duolang Sheep Breeding Center in Maigaiti County, Xinjiang.

RNA extraction

The ovarian tissue stored in the refrigerator at -80°C was taken out, and RNA was extracted using the TransZol Plant kit (Transgen Biotech Co., Ltd., China) according to the manufacturer's instructions.

RNA quality testing

The RNA electropherogram has 3 bands, from top to bottom are 28S, 18S, and 5S rRNA.

Reverse transcription to cDNA

According to the manufacturer's instructions. Reverse transcription was performed by using a PrimeScript RT reagent kit (Takara Bio) with 300 ng RNA in a 10 µl reaction mixture containing 2 µl PrimeScript buffer, 0.5 µl PrimeScript reverse transcriptase enzyme mix I, 0.5 µl oligo(dT) primer (50 µM) and 0.5 µl random hexamers (100 µM). The mixtures were incubated at 37 °C for 40 min to synthesize cDNA and heated to 85 °C for 10 s to inactivate the reverse transcriptase.

Fluorescence quantitative PCR reaction

Using cDNA as a template, real-time fluorescent quantitative PCR was used to detect the expression of the gene to be quantified in each tissue. The reaction system is 5.5µL ddH2O, 7.5µL 2×Transtant qPCR Mix, the upstream and downstream primers are each 0.5µL, 1µL cDNA, and the total reaction system is 15µL. Reaction conditions: 95°C, 2min; 95°C, 15s; 55°C, 15s; 68°C, 20s, repeat the reaction 40 times. This experiment uses the $2^{-\triangle\triangle Ct}$ analysis method.

$$\text{Ratio}_{(test/calibrator)} = 2^{-[\Delta C_T(test)\ -\Delta C_T(calibrator)]}$$
