# Supplementary material for: Analysis on the desert adaptability of indigenous sheep in the southern edge of Taklimakan Desert
Source: Sci Rep. 2022 Jul 18;12:12264. doi: 10.1038/s41598-022-15986-x (PMC9293982; doi:10.1038/s41598-022-15986-x)
Supplement: Supplementary file 3 — Supplementary Legends. [file 41598_2022_15986_MOESM3_ESM.docx]

**Additional information**

**Appendix Table.xlsx**

S1: Information on gene primers involved in the qRT-PCR experiments.

S2: The intersection and union relationship of all the first 1% results of selective cleaning. TRUE means that this group of genes was detected by the current test, while FALSE means that this group of genes was not detected. In order to make the table more concise, we adopt the following rules to correspond to its test, 1: IHS(a); 2: IHS(b); 3: FST(a); 4: FST(b); 5: Rsb(a); 6: Rsb(b); 7: xp-EHH(a); 8: xp-EHH(b).

S3: Top 5% of the results detected by the iHS method for QR genomic selection signal.

S4: Top 5% of the results detected by the iHS method for DL genomic selection signal.

S5: The first 5% difference results in (a) QR and DL with HSK as detected by the FST method.

S6: The first 5% difference results in (b) QR and DL with APD and ASU as detected by the FST method.

S7: The first 5% difference results in (a) QR and DL with HSK as detected by the Rsb method.

S8: The first 5% difference results in (b) QR and DL with APD and ASU as detected by the Rsb method.

S9: The first 5% difference results in (a) QR and DL with HSK as detected by the xp-EHH method.

S10: The first 5% difference results in (b) QR and DL with APD and ASU as detected by the xp-EHH method. **13/19**

S11: Selectively sweeping the GO enrichment results of candidate genes.

S12: Selectively sweeping the KEGG enrichment results of candidate genes.

S13: Selectively sweeping the Reactome enrichment results of candidate genes.

S14: Selectively sweeping the Human Phenotype Ontology (HP) enrichment results of candidate genes.

S15: RNA-Seq results in ovary tissues of Duolang sheep at different stages, while using FPKM to measure gene expression.

S16: Joint analysis of transcription results and genome selection signal results.

S17: Gene union detected by FST, xp-EHH, Rsb and iHS under the threshold of 1%.

**Thesis appendix.pdf**

Scheme and details of qRT-PCR experiment.
